# Disruption of rack1 suppresses SHH‐type medulloblastoma formation in mice

**DOI:** 10.1111/cns.13728

**Published:** 2021-09-04

**Authors:** Fengjiao Liu, Jingyuan Shao, Haihong Yang, Guochao Yang, Qian Zhu, Yan Wu, Lingling Zhu, Haitao Wu

**Affiliations:** ^1^ Department of Neurobiology Beijing Institute of Basic Medical Sciences Beijing China; ^2^ Department of Anesthesiology The General Hospital of Western Theater Command Chengdu China; ^3^ School of Basic Medicine Qingdao University Qingdao China; ^4^ Key Laboratory of Neuroregeneration Co‐innovation Center of Neuroregeneration Nantong University Nantong China; ^5^ Chinese Institute for Brain Research Beijing China

**Keywords:** Gli1, GNPs, medulloblastoma, rack1, Sonic hedgehog

## Abstract

**Introduction:**

Medulloblastoma (MB) is a malignant pediatric brain tumor that arises in the cerebellar granular neurons. Sonic Hedgehog subtype of MB (SHH‐MB) is one of the major subtypes of MB in the clinic. However, the molecular mechanisms underlying MB tumorigenesis are still not fully understood.

**Aims:**

Our previous work demonstrated that the receptor for activated C kinase 1 (Rack1) is essential for SHH signaling activation in granule neuron progenitors (GNPs) during cerebellar development. To investigate the potential role of Rack1 in MB development, human MB tissue array and SHH‐MB genetic mouse model were used to study the expression of function of Rack1 in MB pathogenesis.

**Results:**

We found that the expression of Rack1 was significantly upregulated in the majority of human cerebellar MB tumors. Genetic ablation of Rack1 expression in SHH‐MB tumor mice could significantly inhibit MB proliferation, reduce the tumor size, and prolong the survival of tumor rescue mice. Interestingly, neither apoptosis nor autophagy levels were affected in Rack1‐deletion rescue mice compared to WT mice, but the expression of Gli1 and HDAC2 was significantly decreased suggesting the inactivation of SHH signaling pathway in rescue mice.

**Conclusion:**

Our results demonstrated that Rack1 may serve as a potential candidate for the diagnostic marker and therapeutic target of MB, including SHH‐MB.

## Graphical abstract

Rack1 is essential for GNPs development by activation of Shh signaling. Here, we found that Rack1 was significantly upregulated in some cerebellar MB tumor samples. Genetic ablation of Rack1 in SHH‐subtype medulloblastoma (MB) tumor mice could significantly reduce tumor cell proliferation, reduce the tumor size, and prolong the survival of tumor rescue mice, indicating that Rack1 may serve as a potential novel therapeutic target for SHH‐type MB in the clinic.

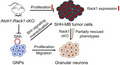

## 1 INTRODUCTION

Medulloblastoma (MB) is the most common childhood malignant tumor in the central nervous system,^1^ which is believed to be led by over‐proliferation of granule cells in the cerebellum. As a developmental tumor, the onset of MB is most common in infants and young children, but also sporadically in adolescents and adults as well. Arising from the cerebellar vermis, MB normally forms along ventricles.^2^ MB is thought to arise from precursors of the granule cells, whose developmental molecules are out of control.^3^ Molecular diagnosis indicated that MB includes four major subgroups: WNT subtype (WNT‐MB), Sonic hedgehog subtype (SHH‐MB), group 3 and group 4 subtypes.^4^ In mice, SHH, as a key mitogen, secreted by Purkinje cells from late embryonic period (~E17) to postnatal period (~P16) stimulates GNPs neurogenesis, which are the basis for the formation of cerebellar lobules.^5^, ^6^, ^7^, ^8^, ^9^, ^10^, ^11^ Deficiency of SHH accounts for about 30% of MB with diverse histopathological and age characteristics^12^, ^13^. Abnormal activation of SHH signaling leads to excessive proliferation of GNPs which will lead to SHH‐MB formation.^8^, ^14^, ^15^ Attempts were carried out recently to recognize possible new therapeutic targets, particularly targeting Smoothened (SMO),^16^, ^17^ which is the upstream regulator of the SHH signaling pathway.^18^ However, despite many targets of MB have been identified, many of those show resistance and progression of disease after the initial inhibitory effect in SHH‐MB.^19^, ^20^

Rack1 (the receptor for activated C kinase 1) is a spheroidal scaffolding protein composed of seven WD40 repeating domains.^21^ The structural characteristics of Rack1 enable it to bind a variety of proteins and play plenty of regulatory functions in multiple cells. According to previous studies, Rack1 has close relevance to tumor formation. Its expression is significantly increased in liver cancer, non‐small cell lung cancer, and glioma, but decreased in gastric cancer and colon cancer.^22^ Rack1 deficiency can inhibit the proliferation and migration of neuroblastoma in vitro.^23^ Nevertheless, the potential function of Rack1 in the cerebellar MB formation remains elusive.

Our previous study has demonstrated that Rack1 is essential for SHH signaling activation in GNPs during postnatal mammalian cerebellar development.^24^ Ablation of Rack1 in GNPs results in a significant deficiency of proliferation and migration in the developing cerebellum,^24^ raising the possibility of whether ablation of Rack1 could suppress SHH‐MB formation. In this study, we firstly observed a significant increase in Rack1 expression in human cerebellar MB tumors. Subsequently, using a SHH‐MB genetic mouse model, *Atoh1*‐*Cre*; *SmoM2* ^+/−^, we demonstrated that ablation of *Rack1* could significantly inhibit the growth of tumor cells with a dramatically extended lifespan in rescue mice. Meanwhile, *Rack1* deficiency did not cause significantly increased cell death owing to apoptosis or autophagy analysis, but dramatically reduced the proliferation of MB tumor cells. Mechanistic studies indicate that the expression of crucial molecules downstream of SHH signaling pathway, including Gli1 and HDAC2, was significantly decreased. These results suggest that the tumor suppression effect in Rack1‐deficient SHH‐MB mice might be related to the reduced hyper‐activation of the SHH signaling pathway. Therefore, our study indicates that Rack1 may serve as a potential candidate for the diagnostic marker and therapeutic target of MB, including SHH‐MB in the clinic.

## 2 MATERIALS AND METHODS

### 2.1 Human MB tissue array

Human paraffin‐embedded tissue array was purchased from Alivabio (DC‐Bra01011b). The patients' sex, age, and histological data were included in Table 1. Tumor tissue array contained 65 points including 20 MB patients (4–49 years old), one healthy human cerebellum, and one patient's adrenal gland. The diameter for each point of tissue is 1.5 mm.

**TABLE 1:** Histological data of human paraffin‐embedded tissue array

Location	Age	Sex	Organ	Pathological diagnosis	Type
I 1	8	M	Cerebellum	Medulloblastoma	Malignant
I 2	8	M	Cerebellum	Medulloblastoma	Malignant
I 3	8	M	Cerebellum	Medulloblastoma	Malignant
I 4	27	F	Cerebellum	Medulloblastoma	Malignant
I 5	27	F	Cerebellum	Medulloblastoma	Malignant
I 6	27	F	Cerebellum	Medulloblastoma	Malignant
I 7	16	M	Cerebrum	Medulloblastoma	Malignant
I 8	16	M	Cerebrum	Medulloblastoma	Malignant
I 9	16	M	Cerebrum	Medulloblastoma	Malignant
II 1	42	M	Cerebellum	Medulloblastoma of vermis cerebellum	Malignant
II 2	42	M	Cerebellum	Medulloblastoma of vermis cerebellum	Malignant
II 3	42	M	Cerebellum	Medulloblastoma of vermis cerebellum	Malignant
II 4	14	M	Cerebrum	Medulloblastoma	Malignant
II 5	14	M	Cerebrum	Medulloblastoma	Malignant
II 6	14	M	Cerebrum	Medulloblastoma	Malignant
II 7	9	M	Cerebrum	Medulloblastoma	Malignant
II 8	9	M	Cerebrum	Medulloblastoma	Malignant
II 9	9	M	Cerebrum	Medulloblastoma	Malignant
III 1	7	M	Cerebellum	Medulloblastoma	Malignant
III 2	7	M	Cerebellum	Medulloblastoma	Malignant
III 3	7	M	Cerebellum	Medulloblastoma	Malignant
III 4	12	F	Cerebrum	Medulloblastoma	Malignant
III 5	12	F	Cerebrum	Medulloblastoma	Malignant
III 6	12	F	Cerebrum	Medulloblastoma	Malignant
III 7	34	M	Cerebrum	Medulloblastoma	Malignant
III 8	34	M	Cerebrum	Medulloblastoma	Malignant
III 9	34	M	Cerebrum	Medulloblastoma	Malignant
IV 1	33	F	Cerebrum	Medulloblastoma of posterior cranial fossa	Malignant
IV 2	33	F	Cerebrum	Medulloblastoma of posterior cranial fossa	Malignant
IV 3	33	F	Cerebrum	Medulloblastoma of posterior cranial fossa	Malignant
IV 4	8	F	Cerebellum	Medulloblastoma	Malignant
IV 5	8	F	Cerebellum	Medulloblastoma	Malignant
IV 6	8	F	Cerebellum	Medulloblastoma	Malignant
IV 7	30	M	Cerebellum	Medulloblastoma	Malignant
IV 8	30	M	Cerebellum	Medulloblastoma	Malignant
IV 9	30	M	Cerebellum	Medulloblastoma	Malignant
V 1	41	M	Cerebellum	Medulloblastoma	Malignant
V 2	41	M	Cerebellum	Medulloblastoma	Malignant
V 3	41	M	Cerebellum	Medulloblastoma	Malignant
V 4	14	F	Cerebellum	Medulloblastoma of vermis cerebellum	Malignant
V 5	14	F	Cerebellum	Medulloblastoma of vermis cerebellum	Malignant
V 6	14	F	Cerebellum	Medulloblastoma of vermis cerebellum	Malignant
V 7	4	F	Cerebrum	Medulloblastoma of vermis cerebellum	Malignant
V 8	4	F	Cerebrum	Medulloblastoma of vermis cerebellum	Malignant
V 9	4	F	Cerebrum	Medulloblastoma of vermis cerebellum	Malignant
VI 1	6	M	Cerebellum	Medulloblastoma of vermis cerebellum	Malignant
VI 2	6	M	Cerebellum	Medulloblastoma of vermis cerebellum	Malignant
VI 3	6	M	Cerebellum	Medulloblastoma of vermis cerebellum	Malignant
VI 4	6	F	Cerebellum	Medulloblastoma	Malignant
VI 5	6	F	Cerebellum	Medulloblastoma	Malignant
VI 6	6	F	Cerebellum	Medulloblastoma	Malignant
VI 7	41	M	Cerebrum	Medulloblastoma of posterior cranial fossa	Malignant
VI 8	41	M	Cerebrum	Medulloblastoma of posterior cranial fossa	Malignant
VI 9	41	M	Cerebrum	Medulloblastoma of posterior cranial fossa	Malignant
VII 1	49	M	Cerebrum	Medulloblastoma of posterior cranial fossa	Malignant
VII 2	49	M	Cerebrum	Medulloblastoma of posterior cranial fossa	Malignant
VII 3	49	M	Cerebrum	Medulloblastoma of posterior cranial fossa	Malignant
VII 4	25	M	Cerebrum	Medulloblastoma of fourth ventricle	Malignant
VII 5	25	M	Cerebrum	Medulloblastoma of fourth ventricle	Malignant
VII 6	25	M	Cerebrum	Medulloblastoma of fourth ventricle	Malignant
VII 7	35	M	Cerebrum	Normal cerebrum tissue	Normal
VII 8	48	M	Cerebrum	Normal cerebrum tissue	Normal
VII 9	24	F	Cerebellum	Normal cerebellum tissue	Normal
–	42	M	Adrenal gland	Pheochromocytoma (tissue marker)	Malignant

### 2.2 Mouse lines

The *Atoh1*‐*Cre*, *Ai9*, *Rack1^F^* ^/^ *^F^*, and *SmoM2* ^+/+^ lines were generated as previously described.^25^, ^26^, ^27^, ^28^, ^29^ Homozygous *Rack1^F^* ^/^ *^F^* mice were crossed with mice expressing transgene encoding *Atoh1*‐*Cre* recombinase and holding *SmoM2* mutation. Conditional knockout (cKO) and rescue mice were generated by the second generation, and *SmoM2* ^+/−^; *Rack1^F^* ^/^ *^F^* littermates were used as controls. Mice were kept in individually ventilated cages on a constant light‐dark rhythm of 12/12 h. Water and food were adequate supply and animals of both sexes were used for the experiments. All experiments with animals were performed in conformity to protocols approved by the Institutional Animal Care and Use Committee of Beijing Institute of Basic Medical Sciences. All of the in vivo animal experiments carried out in this study were in compliance with the ARRIVE 2.0 guidelines.^30^

All mice were genotyped by PCR assay using murine tail DNA. Sequences of the oligo nucleotides used for the genotyping were provided in Table 2.

**TABLE 2:** Oligo sequences for genotyping

Gene	Primer	Primer sequence
*Rack1*:	F	CGCTGCGCCTCTGGGATCTCA
	R	TGGTGTGGCCGACAAATCGCC
*Atoh1*‐*cre*:	F	CCGGCAGAGTTTACAGAAGC
	R	ATGTTTAGCTGGCCCAAATG
*Ai9*:	F1	AAGGGAGCTGCAGTGGAGTA
	F2	GGCATTAAAGCAGCGTATCC
	R1	CCGAAAATCTGTGGGAAGTC
	R2	CTGTTCCTGTACGGCATGG
*SmoM2*:	F1	AAAGTCGCTCTGAGTTGTTAT
	F2	GCGAAGAGTTTGTCCTCAACC
	R	GGAGCGGGAGAAATGGATATG

Note

F, forward primer; R, reverse primer.

### 2.3 Antibodies and reagents

Anti‐β‐actin (1:2000, Sangene biotech, KM9001); anti‐Rack1 (1:2000, Sigma, R1905); anti‐HDAC2 (1:1000, CST, 5113); anti‐Gli1 (1:1000, Sigma, SAB2700185); anti‐NeuN (1:400, Millipore, MAB377); anti‐Ki67(1:400, BD, 550609); anti‐LC3(ABclone, A19665); Alexa Fluor 568‐ or Alexa Fluor 488‐conjugated fluorescent secondary antibody (1:400; Thermo Fisher Scientific); DAPI (H‐1200; VECTASHIELD); anti‐BrdU (abcam, ab6326); Triton X‐100 (Molecular Probe); phosphate buffered saline (PBS, Solarbio); 4% paraformaldehyde (Solarbio, P1110); and bovine serum albumin (BSA; Solarbio, A8010).

### 2.4 Immunofluorescence, Immunohistochemistry, Hematoxylin, and Eosin (H&E) staining

Brain samples were fixed in 4% paraformaldehyde overnight and dehydrated by gradient with 15% and 30% sucrose solution (PBS). And OCT (Tissue‐tek, 4583) was used to embed the brain, and frozen sections were performed. The immunofluorescent staining (IF) of frozen cerebellar sections was performed using standard techniques as previously described.^24^ Briefly, frozen sections (40 μm) were washed for 10 min with 0.3% Triton X‐100/PBS (PBST) for three times and then covered with 3% BSA in PBST (0.3% Triton X‐100) for 1 h. The sections were then incubated with primary antibodies overnight at 4°C. Subsequently, the sections were washed for 10 min with 0.3% PBST for three times followed by Alexa Fluor 568‐ or Alexa Fluor 488‐conjugated fluorescent secondary antibody incubation. Nuclear staining was directly covered with a mounting medium with DAPI. All images were processed and analyzed by using Image Pro Plus software or FV10‐ASW.

For immunohistochemistry staining of human MB tissue array, antibodies against Rack1 were used. And the paraffin‐embedded human MB tissues (Table 2) were incubated with blocking solution including 4% horse serum, 1% BSA, and 0.3% Triton‐X in PBS, and followed by primary antibody incubation overnight at 4°C. Then, slides were incubated with horseradish peroxidase (HRP)‐conjugated secondary antibodies for 45 min at room temperature. The sections were then washed for 10 min with 0.3% PBST for three times followed by incubation with 3,3′‐diaminobenzidine (DAB) solution for a few minutes. Subsequently, the sections were rinsed in PBS and mounted with the mounting medium.

The H&E staining of frozen cerebellar sections was performed using standard techniques as previously described.^24^ Briefly, slides were stained in hematoxylin solution for 8 min, then wash in running tap water for 5 min. Subsequently, differentiate in 1% acid alcohol for 30 s and wash running tap water for 1 min. Counterstain in eosin‐phloxine solution for 30 s to 1 min, and dehydrate through 95% alcohol, two changes of absolute alcohol, 5 min each. Finally, the slides were cleared in two changes of xylene and mounted with xylene‐based mounting medium.

### 2.5 5‐Bromo‐2'‐deoxyuridine incorporation and in vivo labeling

5‐Bromo‐2'‐deoxyuridine (BrdU; 10 mg/ml; Sigma, B5002) solution was dissolved in PBS. The solution was always kept out of light and stable at −20°C for about 6 months. In this study, P29 mice were used for BrdU incorporation assay, 24 h after weighing mice and intraperitoneal injection of BrdU solution at a dose of 50 mg/kg, the mice were euthanized by cervical dislocation. Then fix and process brain tissue for DNA hydrolysis, which is essential for BrdU staining before continuing with the immunostaining step. Briefly, cerebellar sections were incubated in 1 M HCl for 30 min at room temperature. The sections were then neutralized by incubating in 0.1 M sodium borate buffer (pH 8.5) for 10 min at room temperature. After three times wash in PBS, continue with standard immunohistochemistry protocol as described above.

### 2.6 Western blot analysis

The experiments were performed as previously described.^24^ Briefly, cerebellar tissues were lysed by RIPA lysis and extraction buffer (89901; Thermo), supplemented with 1× protease inhibitor mixture (A32965; Thermo) and phosphatase inhibitor mixture, in an ice bath for 20 min. Protein concentration was measured using the BCA Protein Assay Kit (23227; Thermo). To identify the protein levels of the lysates, samples (20–50 μg) were carried out by using sodium dodecyl sulfate‐polyacrylamide gel electrophoresis (SDS‐PAGE) and transferred to polyvinylidene fluoride (PVDF) membranes (10600023; General Electric). The membranes were then blocked with 5% skim milk in 0.1% Tris‐buffered saline/Tween 20 for 1 h and incubated overnight at 4°C with primary antibodies. The film signal was electronically scanned and analyzed by Image Pro Plus.

### 2.7 RNA extraction, reverse transcription, and quantitative real‐time PCR analysis

TRIzol reagent (Invitrogen, 15596–026) was used to extract RNA from the cerebellum of mice. The total RNA concentration and purity were measured by the Nanodrop 1000^™^ (Thermofisher Scientific) and cDNA was synthesized using M‐MLV (Vazyme, R223‐01), according to the manufacturer's instructions. Quantitative real‐time PCR (RT‐PCR) was performed using Power SYBR Green Master Mix (Cwbio, cw2601) on the Applied Biosystems ViiA 7 real‐time PCR system (Thermo Fisher Scientific), and analyzed using Viia 7 software (Thermo Fisher Scientific). The primers used for RT‐PCR analysis in this study were provided in Table 3.

**TABLE 3:** Oligo sequences for qRT‐PCR

Gene	Primer	Probes
Cdk4	F	ATGGCTGCCACTCGATATGAA
	R	TCCTCCATTAGGAACTCTCACAC
Ccnb1	F	AAGGTGCCTGTGTGTGAACC
	R	GTCAGCCCCATCATCTGCG
Ccnd3	F	TGGATCGCTACCTGTCCTG
	R	CCTGGTCCGTATAGATGCAAAG
Ccne2	F	ATGTCAAGACGCAGCCGTTTA
	R	GCTGATTCCTCCAGACAGTACA
E2f7	F	GCATACGGCCAGATCCGAG
	R	GACCCTTGTCTTTCTCCCTGT
Cdkn2c	F	CCTTGGGGGAACGAGTTGG
	R	AAATTGGGATTAGCACCTCTGAG
GFAP	F	AGGTCGGTGTGAACGGATTTG
	R	TGTAGACCATGTAGTTGAGGTCA

Note

F, forward primer; R, reverse primer.

### 2.8 Statistical analysis

The cerebellar immunofluorescence images were taken from 7 × 7 slices for confocal scanning or imaged with 20× (zoom 2.0) objective multiple‐layer scans. Western blot results and cerebellar section areas were statistically analyzed using GraphPad Prism 8.0 and NDP view. The data between the two independent groups were shown as mean ± SEM for at least three independent experiments. The experimental data were analyzed using the non‐parametric *t*‐test. *p* < 0.05 (*); *p* < 0.01(**); *p* < 0.001(***), or *p* < 0.0001 (****) was considered a statistically significant difference.

## 3 RESULTS

### 3.1 Upregulated expression of Rack1 in some MB samples

**FIGURE 1:**
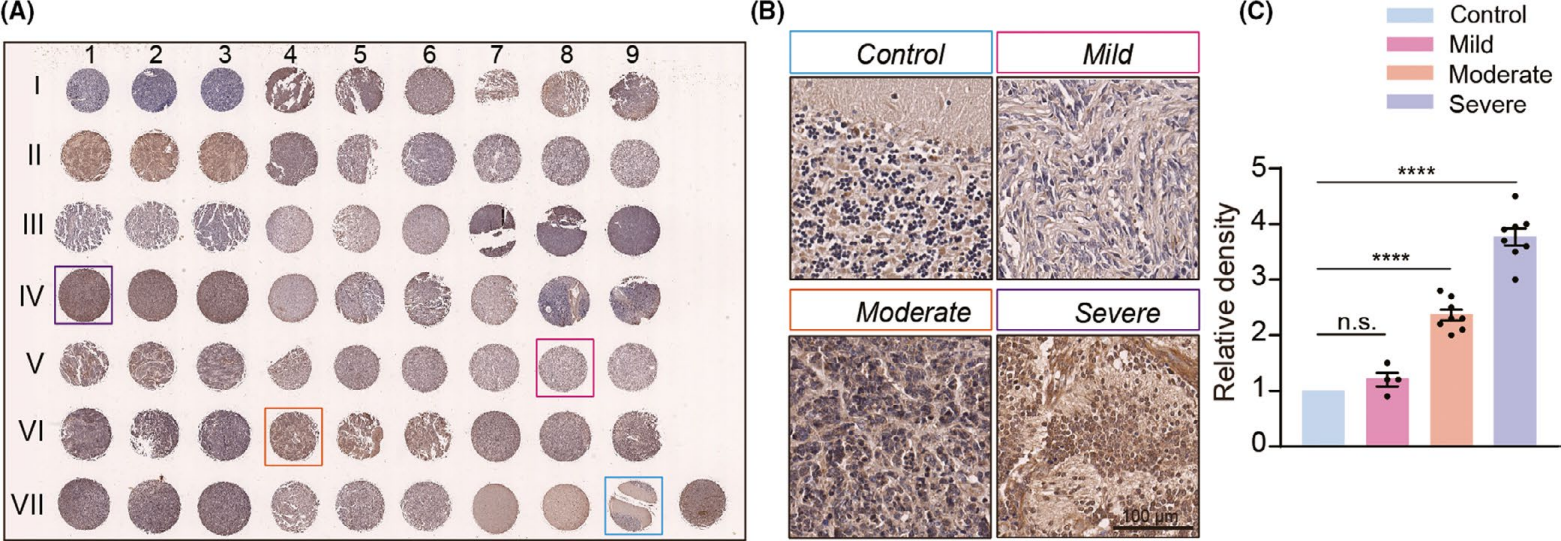
Increased expression of Rack1 in human medulloblastoma samples. (A) Paraffin‐embedded tissue array from human MB samples. The tissues in VII 7–9 are normal human cerebellum as control, and the sample in the lowest right corner is from pheochromocytoma in adrenal gland. (B) Different expression levels of Rack1 in various human MB samples. The expression level of Rack1 was divided into three levels including mild, moderate, and severe compared to normal control cerebellum. VIII 9, control; V 8, mild; VII 4, moderate; IV 1, severe. Scale bar = 100 μm. (C) Quantitative analysis of the relative density of Rack1 expression in different human MB samples (mean ± SEM). ****p < 0.0001, *n* > 3

Our previous studies have demonstrated that Rack1 plays critical roles in regulating granule neuron precursors (GNPs) proliferation and migration during mammalian cerebellar development.^24^ However, whether the expression of Rack1 is aberrantly regulated in clinical MB tumors is still elusive. To address this issue, we first examined the expression of Rack1 in a paraffin‐embedded MB tumor tissue organized row from 20 patients and a normal cerebellum control (Table 1, Figure 1A) by immunohistochemistry staining. We found that 16 out of 20 MB tumors show significantly upregulated expression of Rack1 in comparison to healthy control tissue (Figure 1A).

Next, according to the different expression level of Rack1 in 20 divergent samples, the MB tumors were divided into three subgroups. Four of 20 MB tumors showing weak upregulation of Rack1 (less than two folds, comparing with normal control) were considered as a mild group. Another eight of 20 MB tumors showing moderate upregulation of Rack1 (2–3 folds, comparing with normal control) were considered as a moderate group. The last eight of 20 MB tumors showing dramatic upregulation of Rack1 (>3 folds, comparing with normal control) were considered as a severe group (Figure 1B,C). Therefore, in our case, about 80% MB patients showed significantly upregulated expression of Rack1 compared to normal cerebellar cortex. It should be noted that we cannot group the 20 MB samples according to the typical four types of molecular classification including WNT‐MB, SHH‐MB, group 3, and group 4 due to lack of detailed information of molecular diagnosis and classification for those MB samples. However, together with the critical role of Rack1 in regulating GNPs proliferation and migration,^24^ the dramatically upregulated expression of Rack1 in 80% MB tumors indicates the potential function of Rack1 in the pathogenesis of MB formation.

### 3.2 Ablation of *Rack1* inhibits SHH‐MB formation in SmoM2‐mutant mice

To further address the effect of Rack1 on the pathogenesis of MB development, *Rack1* gene was specifically ablated in GNPs in constitutively activated smoothened (SmoM2) mice by Cre‐mediated recombination. The engineered transgenic mouse line (*SmoM2*) was used in which expression of an inserted dominant active allele of *SmoM2* gene is blocked by a loxP‐flanked stop signal.^31^ Firstly, the homozygous *SmoM2* ^+/+^ mice were crossed to *Rack1^F^* ^/^ *^F^* mice^28^ to get *SmoM2* ^+/−^; *Rack1^F^* ^/^ *^W^* mice. Homozygous *Rack1^F^* ^/^ *^F^* mice were crossed to *Atoh1*‐*Cre* mice in which *Cre* recombinase sequence was inserted into the *Atoh1* gene as previously reported^24^, ^32^ to get mice. Thereafter, *Atoh1*‐*Cre*; *Rack1^F^* ^/^ *^W^* mice were crossed to *SmoM2* ^+/−^; *Rack1^F^* ^/^ *^W^* mice to generate the following four genotypes including: *SmoM2* ^+/−^; *Rack1^F^* ^/^ *^F^* (indicated as WT mice), *Atoh1*‐*Cre*; *Rack1^F^* ^/^ *^F^* (cKO mice); *Atoh1*‐*Cre*; *SmoM2* ^+/−^ (indicated as SHH‐MB mice) and *Atoh1*‐*Cre*; *SmoM2* ^+/−^; *Rack1^F^* ^/^ *^F^* (indicated as Rescue mice).

**FIGURE 2:**
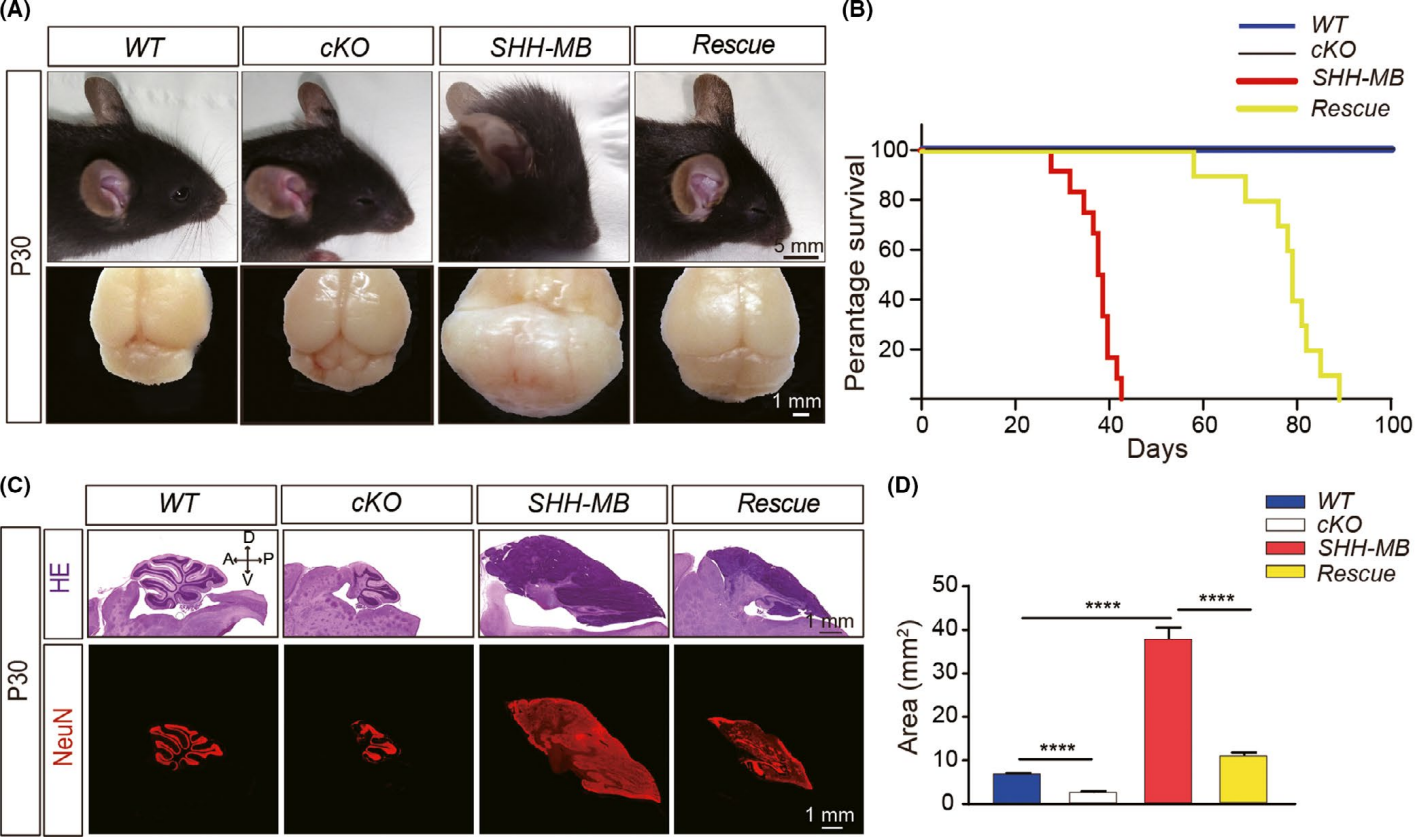
Ablation of Rack1 in SHH‐MB mice suppresses MB tumorigenesis. (A) Gross morphology of P30 cerebellums in *SmoM2* ^+/−^; *Rack1^F^* ^/^ *^F^* control (WT), *Atoh1*‐*Cre*; *Rack1^F^* ^/^ *^F^* conditional knockout mice (cKO); *Atoh1*‐*Cre*; *SmoM2* ^+/−^ tumor mice (SHH‐MB), and *Atoh1*‐*Cre*; *SmoM2* ^+/−^; *Rack1^F^* ^/^ *^F^* rescue mice (Rescue). The volume of the tumor cerebellum in rescue mice was significantly reduced compared to SHH‐MB tumor mice. Scale bar = 1 cm (top panel) and 1 mm (bottom panel), respectively. (B) Survival curves between different genotypes. WT: *n* = 13; cKO: *n* = 14; SHH‐MB: *n* = 12; Rescue: *n* = 10. (C) HE staining and immunofluorescent staining with anti‐NeuN of sagittal histological sections of the cerebellar vermis in different genotypes at P30. Scale bar = 1 mm. (D) Quantitative analysis of sagittal area of vermis sections in different genotypes at P30 indicating the significantly decreased area of vermis sections in rescue mice compared to SHH‐MB tumor mice. ****p < 0.0001, *n* ≥ 3

Further analysis demonstrated that, in terms of overall gross morphology of the cerebellum in different genotypes, the vermis area in the sagittal section of cerebellum in SHH‐MB mice were dramatically increased compared to WT mice (the coronal section of the vermis was 7.07 ± 0.05 mm^2^ in WT, and 11.04 ± 0.52 mm^2^ in SHH‐MB mice, *p* < 0.0001, *n* = 6; Figure 2A‐D). In contrast, in agreement with our previous studies,^24^ the vermis size of the cKO cerebellum was significantly reduced compared to WT mice at P30 (7.07 ± 0.05 mm^2^ in WT *vs*. 2.75 ± 0.17 mm^2^ in cKO mice, *p* < 0.0001, *n* = 6; Figure 2A‐D). Interestingly, both the total volume and the vermis size of the cerebellum were significantly reduced in rescued mice compared to SHH‐MB mice (11.04 ± 0.52 mm^2^ in rescue mice *vs*. 37.94 ± 2.54 mm^2^ in SHH‐MB mice, *p* < 0.0001, *n* = 7), although the foliation patterning of cerebellar cortex in rescued mice can hardly be identified (Figure 2C). It should be noted that the malignant MB tumor cells in SHH‐MB cerebellum were NeuN+cells, indicating that they are granular neurons in the cerebellum. In addition to the significantly reduced size of MB tumor in rescued mice, our data further shows that, consistent with HE staining, the sagittal area shown by NeuN+cells in the cerebellum was also significantly reduced in rescue mice (Figure 2C). Moreover, the MB tumor growth rescue effect was more significant at the rostral area compared to the caudal region, which might be due to the distinct expression pattern of *Atoh1*‐*Cre* as previously described^24^ (Figure S1).

To study the beneficial effect of Rack1 deletion on MB tumor therapy, we further compared the survival curve between different genotypes. In contrast to the relatively normal survival rate of *Rack1* cKO mice compared to WT mice, the lifespan of SHH‐MB mice was dramatically decreased for a period of 28–43 days, by the point of day 43, the survival of the WT, cKO, and the rescued mice was 100% (13 of 13, 14 of 14, and 10 of 10, respectively), whereas the survival of SHH‐MB was 0% (12 of 12; Figure 2B). Accordingly, we showed that the average lifespan of the rescued mice model was prolonged over 2‐fold (range from 58 to 89 days; Figure 2B). Together, these results indicated that suppression of Rack1 expression in GNPs could significantly inhibit SHH‐MB tumor growth and increased the survival rate of rescued mice.

### 3.3 Ablation of *Rack1* suppresses MB proliferation instead of induction of the cell death

**FIGURE 3:**
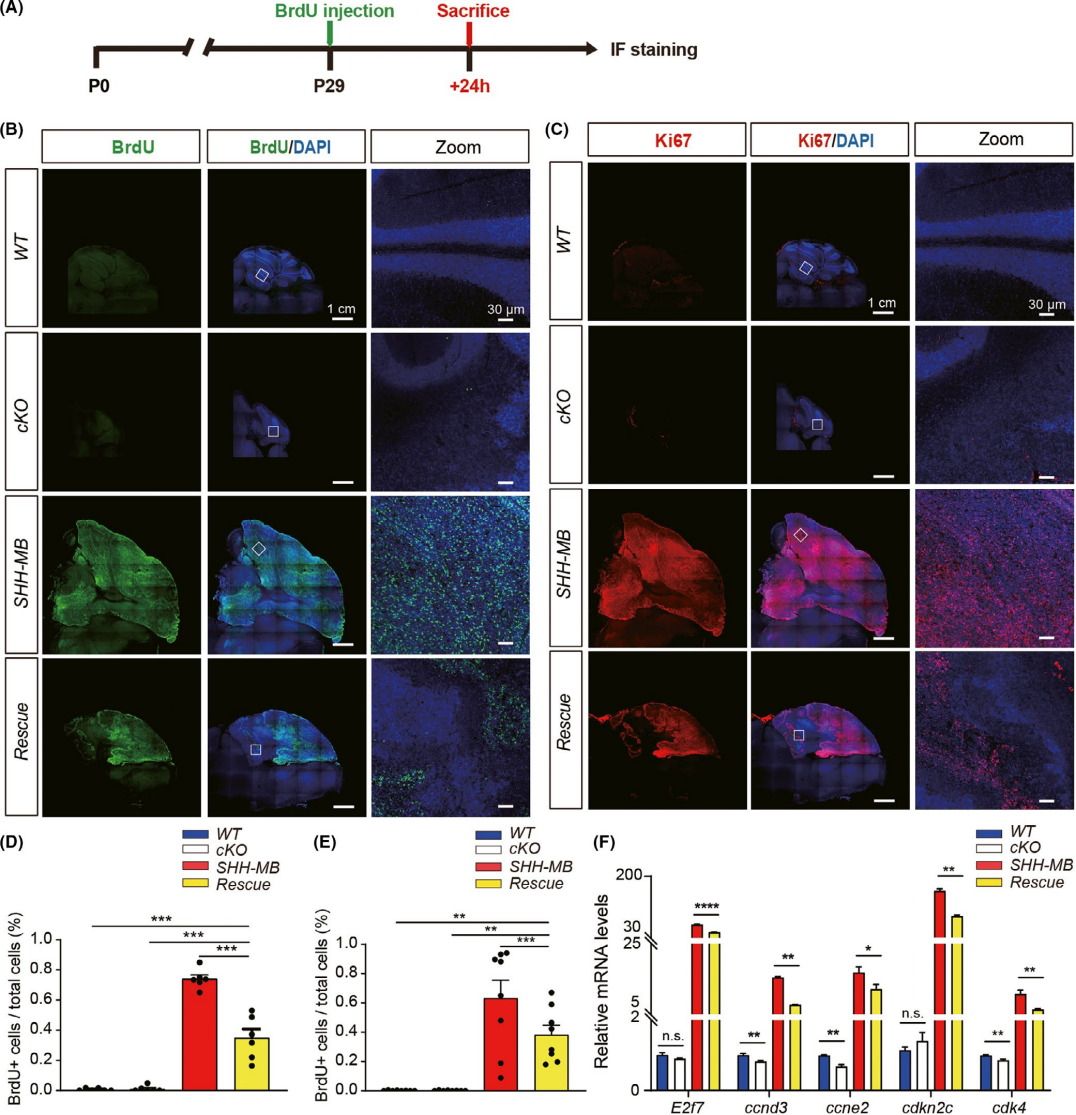
Loss of Rack1 suppresses the proliferation of MB tumor cells. (A) Schematic diagram showing the proliferation of granular neurons was evaluated by BrdU administration and incorporation assay. (B and C) Proliferating granular neurons were immunolabeled with anti‐BrdU and anti‐Ki67 antibodies, respectively. (D and E) Quantitative analysis of proliferating granular neurons shows significantly decreased percentage of BrdU^+^(C) and Ki67^+^(E) cells in rescue mice compared with SHH‐MB tumor mice. **p < 0.01, ***p < 0.001, *n* ≥ 3. (F) Quantitative RT‐PCR (qRT‐PCR) analysis shows significantly downregulated expression of *E2f7*, *ccnd3*, *ccne2*, *cckn2c* and *cdk4* in rescue mice compared to SHH‐MB tumor mice. *p < 0.05, **p < 0.01, ***p < 0.001, ****p < 0.0001, n.s., not significant, *n* ≥ 3

To further investigate how Rack1 deletion causes the inhibition of SHH‐MB tumor formation, we compared the proliferation and cell death of SHH‐MB tumor cells in vivo. Firstly, BrdU incorporation assay was carried out to investigate the proliferating MB tumor cells, especially at S‐phase. BrdU was intraperitoneally injected into mice at P29, and 4% paraformaldehyde was infused into the mice 24 h later (Figure 3A). Immunofluorescence staining results showed that a large population of BrdU+proliferative cells were widely distributed in SHH‐MB tumor mice model at P30, a non‐proliferation stage of cerebellar granule neurons in WT mice (Figure 3B). In contrast, the number of BrdU+proliferative cells was significantly reduced in the rescued mice (Figure 3B,D). Similar results were observed by Ki67 immunofluorescent staining in SHH‐MB tumor and rescue mice models at P30 (Figure 3C,E), further indicating that deletion of Rack1 could significantly suppress the proliferation of SHH‐MB tumor cells.

In addition, we also check the transcriptional level of several proliferative cell cycle markers including *cdk4*, *ccnb1*, *ccnd3*, *ccne2*, *E2f7*, and *cdkn2c* in total cerebellar RNA extraction by quantitative RT‐PCR. We found that in the absence of Rack1 in either GNPs (cKO mice) or SHH‐MB tumor cells (Rescue mice), the transcription of *ccnd3* (*p* = 0.0087, *n* = 5; and *p* = 0.0022, *n* = 5, respectively), *ccne2* (*p* = 0.0021, *n* = 5; and *p* = 0.0381, *n* = 5, respectively), and *cdk4* (*p* = 0.0065, *n* = 6; and *p* = 0.0033, *n* = 6, respectively) were all significantly decreased (Figure 3F). Although the expression of *E2f7* and *cdkn2c* was indistinguishable between WT and cKO mice (*p* = 0.180, *n* = 5; and *p* = 0.329, *n* = 4, respectively), both of which were significantly reduced in rescued mice compared to SHH‐MB mice (Figure 3F). These results suggest that Rack1 deficiency in SHH‐MB mice could cause the cell cycle arrest of tumor cells.

**FIGURE 4:**
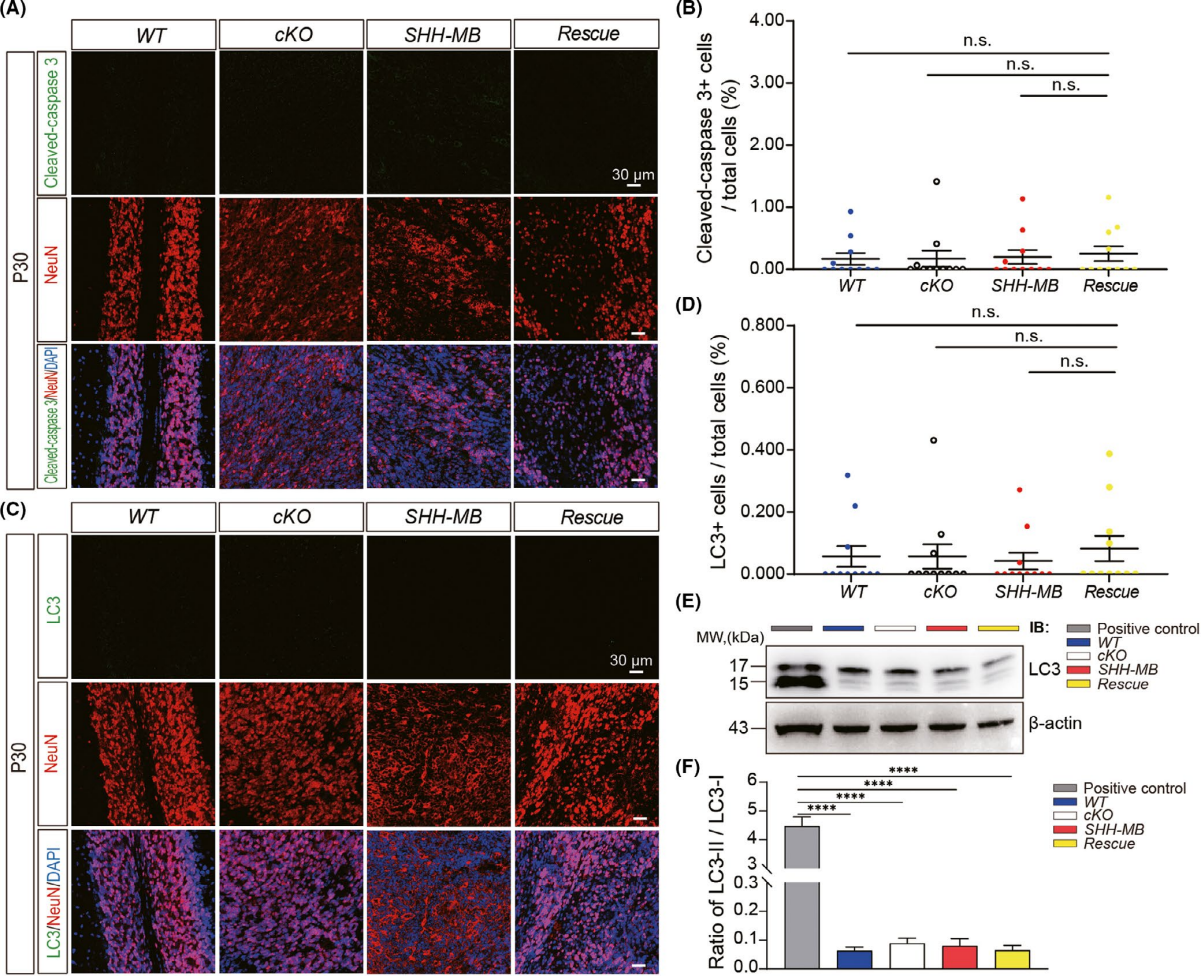
Ablation of Rack1 in SHH‐MB tumor cells does not induce apoptotic effect. (A) Immunofluorescent staining of sagittal histological sections of the P30 vermis in different genotypes with anti‐NeuN and anti‐cleaved‐caspase 3 antibodies. Scale bar = 30 μm. (B) Quantitative analysis shows no significant increase in cleaved‐caspase3^+^ cells. mean ± SEM, n.s., not significant. (C) Immunofluorescent staining of sagittal histological sections of the P30 vermis in different genotypes with anti‐NeuN and anti‐LC3. Scale bar = 30 μm, n.s., not significant. (D) Quantitative analysis shows no significant increase in LC3^+^ cells. mean ± SEM, n.s., not significant. (E) Representative Western blots show the expression of LC3 in cerebellar lysates from different genotypes. SH‐SY5Y cell lysate (treated with 100 μM of OSMI for 24 h) was used as a positive control. (F) Quantitative Western blot analysis indicates in contrast to positive control, no significant increase in the ratio of LC3‐II/LC3‐I between different genotypes. mean ± SEM, ****p < 0.0001, *n* ≥ 3

Next, we asked whether Rack1 deficiency could induce the cell apoptosis of MB tumor cells, which may also contribute to the tumor suppression effect in rescue mice. To test this hypothesis, we performed immunofluorescent staining of the cerebellar vermis sections using anti‐cleaved‐caspase 3 antibodies, a typical marker of the activated apoptotic pathway. However, we found that there was no significantly increased apoptotic cells in either SHH‐MB or Rack1 deletion rescued mice compared to WT (*p* = 0.8321, *n* = 4; and *p* = 0.5877, *n* = 4, respectively) or cKO (*p* = 0.8741, *n* = 5; and *p* = 0.6565, *n* = 4, respectively) mice (Figure 4A,B). Given that the elevated levels of autophagy may also contribute to the cell death of tumor cells,^33^ we further examined the autophagy levels in the cerebellum in distinct genotypes by immunofluorescence staining with anti‐LC3 antibodies, a typical indicator of autophagy processing. Furthermore, there was no significant upregulation or aggregation of LC3 in either SHH‐MB or Rack1 deletion rescued mice compared to WT (*p* = 0.7315, *n* = 4; and *p* = 0.6363, *n* = 4, respectively) or cKO mice (*p* = 0.7572, *n* = 4; and *p* = 0.6617, *n* = 4, respectively), indicating the relatively normal autophagy levels in both SHH‐MB and rescue mice (Figure 4C,D). To avoid the possibility of false‐negative result with immunofluorescence staining, SH‐SY5Y cells were treated with either H_2_O_2_ or OGT inhibitor OSMI to induce apoptosis and autophagy respectively, which show strong immunofluorescence staining results (Figure S2). Moreover, we further used Western blot to probe for expression changes of LC3, a typical autophagy marker, in OSMI‐treated SH‐SY5Y and cerebellum lysates from distinct genotypes, which also shows the cleaved LC3‐II is indistinguishable among all genotypes in contrast to the significantly increased expression in SH‐SY5Y cell lysates (Figure 4E,F). Together, these results further indicate that the negative immunofluorescence staining results in distinct genotypes are bona‐fide.

To further corroborate our conclusion, we carried out electron microscope (EM) analysis to investigate the ultrastructure of MB tumor cells in both SHH‐MB and Rack1 deletion rescued mice. Indeed, there was no obvious increase in either apoptotic body or autophagosome in SHH‐MB and rescue mice (Figure S3). Interestingly, we found a significantly increased number of mitochondria in granular cells in rescue mice. However, almost no mitochondria could be found in SHH‐MB tumor cells (Figure S3). Taken together, these results indicate that deletion of Rack1 in SHH‐MB tumor cells could significantly inhibit the proliferation of MB tumor cells rather than the induction of the MB tumor cell death.

### 3.4 Rack1 deficiency in MB causes the inactivation of the SHH signaling

**FIGURE 5:**
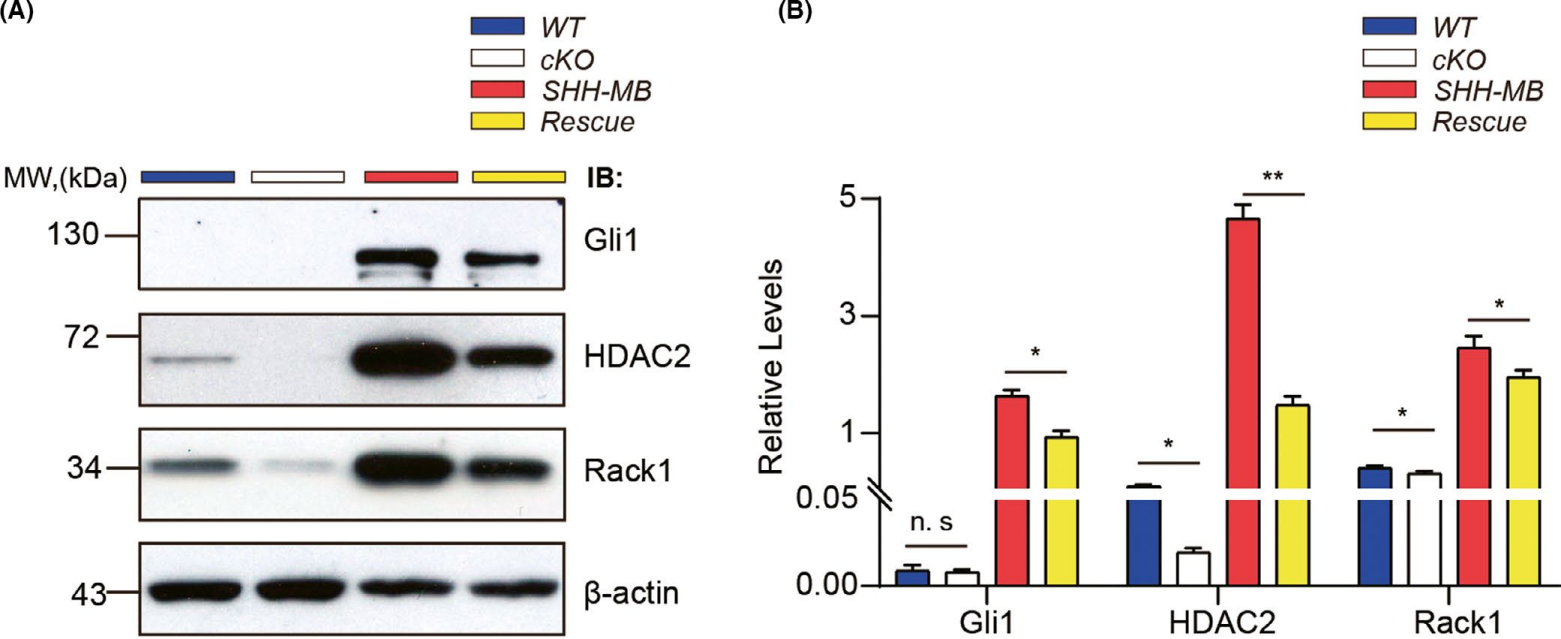
Rack1 deficiency in SHH‐MB inhibits SHH signaling pathway. (A) Western blots detected the expression of Gli1 and HDAC2 in cerebellar lysates from different genotypes. (B) Quantitative analysis of Western blot results indicates the significantly reduced expression of Gli1 and HDAC2 in rescue mice compared to SHH‐MB tumor mice. mean ± SEM; *p < 0.05, **p < 0.01, *n* ≥ 3

Given that SHH signaling was significantly suppressed in Rack1‐deficient GNPs,^24^ and deletion of Rack1 in SHH‐MB could significantly suppress the proliferation of tumor cells (Figure 2), therefore, we asked whether the absence of Rack1 in MB tumor cells could also induce the inactivation of SHH signaling. To address this question, we examined the expression of Gli1 and HDAC2 by Western blot, which are critical activators of SHH signaling pathways. We found that the expression of both Gli1 and HDAC2 was significantly upregulated in SHH‐MB tumors compared to WT cerebellum, indicating the over activation of SHH signaling in SHH‐MB tumor cells. However, the expression of Gli1, HDAC2, and Rack1 was all significantly reduced in rescue mice compared to SHH‐MB mice (Figure 5A,B). These results suggest that Rack1 deficiency could significantly inhibit SHH‐MB tumor growth probably by inactivation of SHH signaling pathway. Our data also suggest that Rack1 is not only essential for GNPs proliferation and migration at postnatal cerebellar developmental stage, which may also play an important role in enhancing SHH‐type MB tumor formation by positively regulating the expression and function of Gli1 and HDAC2.

## 4 DISCUSSION

Medulloblastoma (MB) is one of the most frequent malignant brain tumors in children,^1^ but the genetic and molecular bases of MB remain unclear. About 5% of malignant MB patients have been recently identified with pathogenic germline variants in cancer predisposition genes.^34^ Previous studies have shown that *Rack1* mutations are commonly observed with high frequency in human solid tumors.^22^ However, whether Rack1 is involved in the pathogenesis of MB is still unknown. Our previous studies have shown that Rack1 positively regulates the proliferation and migration of GNPs during cerebellar development by activating SHH signaling pathways.^24^ In this study, we found that the expression of Rack1 was significantly upregulated in the majority of MB tumor samples (Figure 1), suggesting the pathogenic potential of Rack1 in MB development. Interestingly, we also noticed that the expression of *Rack1* gene has been identified with significant upregulation in SHH, WNT, and Type 3 MB tumor samples, but reduction in Type 4 MB tissues based on gene profiling assay using Gene Expression Omnibus (GEO) DataSet (GSE124814) (Figure S4), which was in agreement with our immunostaining results (Figure 1). Together, these results suggest that the abnormal overexpression of Rack1 may be closely related to the human MB tumorigenesis.

Constitutive expression of dominant active *SmoM2* in the GNPs induced SHH‐MB tumor formation.^13^, ^26^ A number of genetically modified mice have been generated previously in which MB development is impaired, and those studies have uncovered important information about the signaling mechanisms involved in SHH‐MB pathogenesis.^35^, ^36^, ^37^, ^38^, ^39^ However, the crucial points affected that directly prevent MB development have remained not fully determined. In this study, the *Rack1*‐deleted SHH‐MB rescue mice provide a useful model with which to directly test the tumor suppression effects on SHH‐MB development. Our results showed a significant reduction in proliferation of MB tumor cells with Rack1 deficiency specifically in GNPs in vivo (Figure 2 and Figure 3), suggesting the effect of Rack1 deletion on MB tumor proliferation might be cell autonomous.

Tumorigenesis can be regulated by the balance of proliferation, differentiation, and apoptosis.^40^ Our studies also showed the significantly downregulated expression of mitotic cell cycle markers Ki67 and BrdU in *Rack1*‐deleted SHH‐MB rescue mice (Figure 3). However, the expression of apoptotic marker Cleaved‐caspase3 and the autophagosomal marker LC3 was virtually undetectable in both SHH‐MB tumor and *Rack1*‐deleted SHH‐MB rescue mice (Figure 4). These results identify a critical role for Rack1 in regulating ectopic proliferation of SHH‐MB tumor cells in vivo. Previous studies demonstrate that Rack1 has a dual role in regulating tumor cell proliferation, apoptosis, and metastasis.^22^ Numerous studies have shown that Rack1 was anomalously expressed in multiple types of human cancers including breast cancer, lung cancer, colorectal cancer, and liver cancers.^28^, ^41^, ^42^, ^43^, ^44^ In contrast to the proapoptotic effect by suppressing Src activity through the intrinsic apoptosis and Akt pathways in colorectal tumorigenesis,^45^ no apoptotic effects have been detected in Rack1 deficiency‐rescued MB tumor mice in this study, suggesting the distinct signaling mechanisms may be mediated by *Rack1* during the tumorigenesis of MB. Therefore, the roles and the underlying signaling mechanisms of Rack1 in different types of human cancers warrant further investigation.

Our previous studies have reported that Rack1 plays a crucial role in regulating the proliferation and migration of GNPs by inhibiting the degradation of HDAC1/2 and therefore promoting the activation of Gli1/2 during postnatal cerebellar development.^24^ It has also been demonstrated that Rack1 promotes non‐small‐cell lung cancer (NSCLC) tumorigenicity by activating Smoothened to mediate Gli1‐dependent transcription in cancer cells.^46^ In this study, we also found that the expression of HDAC2 and Gli1 were both significantly downregulated in rescue mice model, further indicating the crucial role of Rack1‐mediated SHH signaling activation in promoting SHH‐type MB tumorigenesis. In addition, we also found the significantly downregulated expression of *cdk4* in both Rack1 cKO and rescue mice. In agreement with our studies, previous studies have also shown that SHH signaling drives MB growth via CDK6, and inhibition of CDK4/6 significantly attenuates the growth of SHH‐MB.^47^

Moreover, previous studies have identified multiple mutant genes involved in brain tumor pathogenesis.^48^, ^49^, ^50^, ^51^ Given that the significantly upregulated expression of Rack1 in different subtypes of MB tumors based on our immunostaining and gene profiling assay using GEO DataSet (Figure 1 and Figure S4), it would be interesting to ask whether *Rack1* gene mutations are involved in the pathogenesis of brain tumors. Interestingly, according to the Cancer Genome Atlas (TCGA) database, we found that the gene mutation rate of *Rack1* in brain tumor samples was about 15.38% (8/52) based on the gene profiling assay (data not shown). These results indicated that Rack1 mutagenesis might be involved in the pathogenesis and progression of several types of brain tumors, such as gliomas and neuroblastoma,^52^, ^53^ therefore, more detailed mutation sites and the underlying signaling mechanisms of Rack1 in brain tumors need further characterization and investigation.

In conclusion, we demonstrated that the levels of Rack1 were upregulated in majority of MB tumor samples, and genetic ablation of Rack1 in SHH‐MB tumor mice significantly reduced MB proliferation, reduced the tumor size, and prolonged the survival of tumor rescue mice. We propose that Rack1‐mediated Gli1 signaling and cell cycle activation in MB tumor cells might be essential for SHH‐MB tumorigenesis. Rack1 could serve as a potential diagnostic marker and therapeutic target for MB patients in clinic.

## CONFLICT OF INTEREST

The authors declare no conflict of interest.

## AUTHOR CONTRIBUTIONS

Conception and design of the study: F.L. and H.W. Performance of experiments: F.L., J.S., H.Y., G.Y., and Y.W. Analysis and interpretation of the data: F.L., J.S., G.Y., Q.Z., L.Z., and H.W. Manuscript writing: F.L., J.S., and H.W.

## Supporting information

Fig S1‐S4

Fig S4

Fig S5

## Data Availability

The data that support the findings of this study are available from the corresponding author upon reasonable request.
