# Supplementary material for: Disruption of rack1 suppresses SHH‐type medulloblastoma formation in mice
Source: CNS Neurosci Ther. 2021 Sep 4;27(12):1518–30. doi: 10.1111/cns.13728 (PMC8611787; doi:10.1111/cns.13728)
Supplement: Supplementary file 1 — Fig S1‐S4 [file CNS-27-1518-s001.pdf]

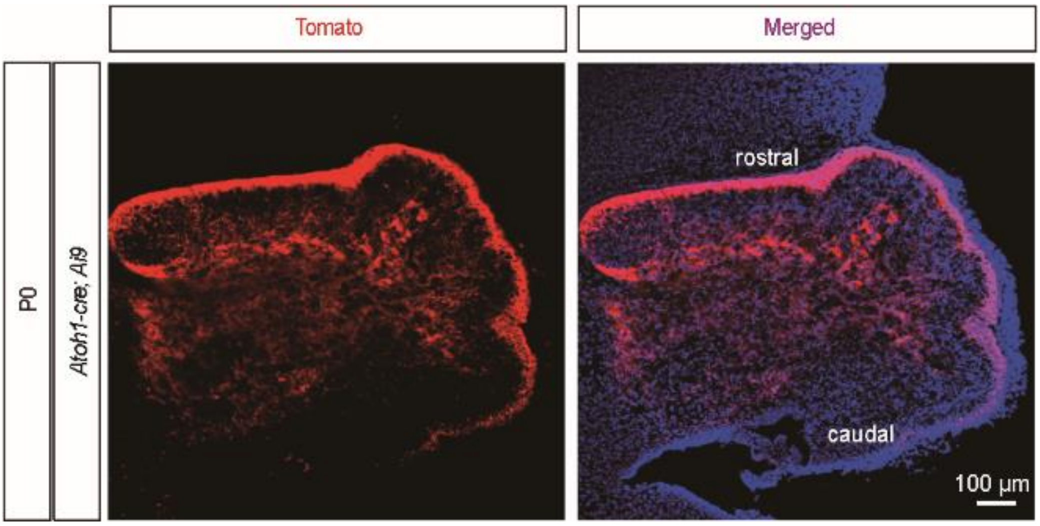

Fig. S1 The expression pattern of Atoh1-Cre recombinase in the cerebellum was indicated by Tomato fluorescence (red) in *Atoh1-cre; Ai9* mice. Scale bar = 100 µm.

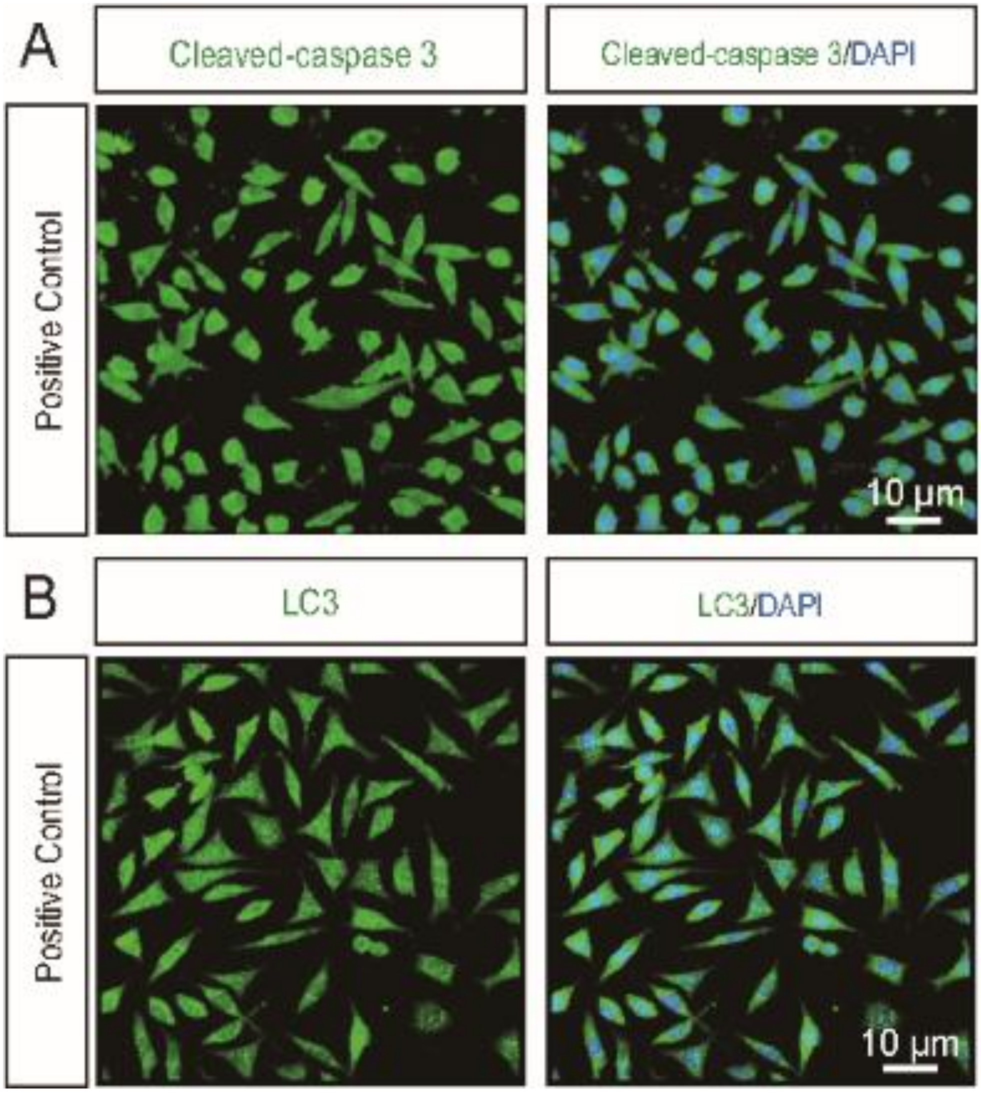

Fig. S2 Immunofluorescent staining of apoptosis and autophagy in SH-SY5Y cells.

(A) Immunofluorescent staining of positive control (SH-SY5Y cells treated with 1mM of H_2O_2 for 24h) with anti-cleaved-caspase3. Scale bar = 10 μm.

(B) Immunofluorescent staining of positive control (SH-SY5Y cell line treated with 100μM of OSMI for 24h) with anti-LC3. Scale bar = 10 μm.

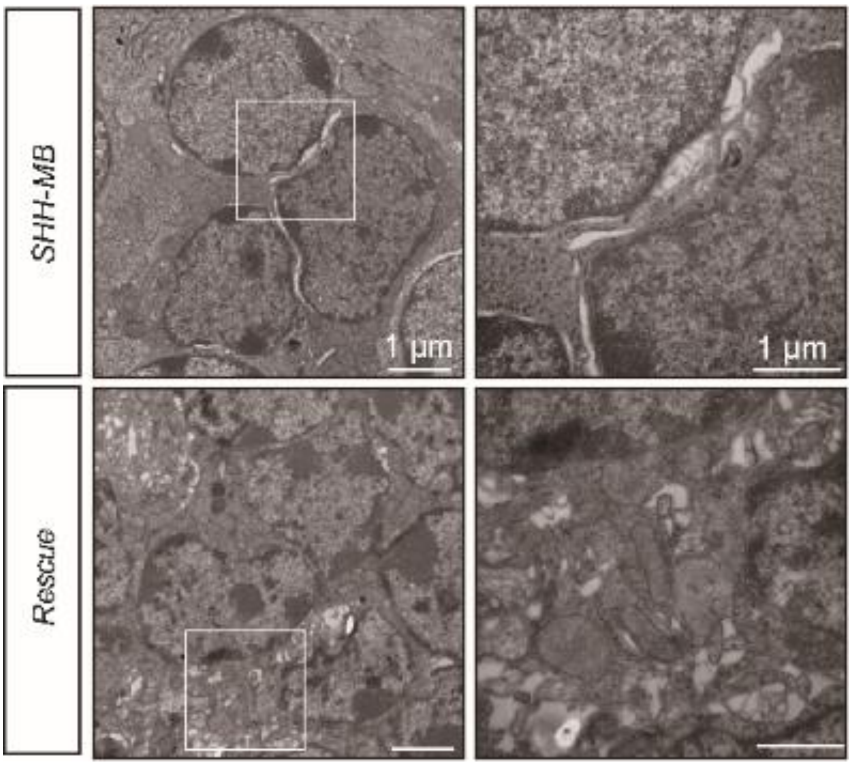

Fig. S3 Electron microscope images indicate subcellular ultrastructure of granular neurons in SHH-MB tumor mice and rescue mice at P30. Scale bar = 1 μm.

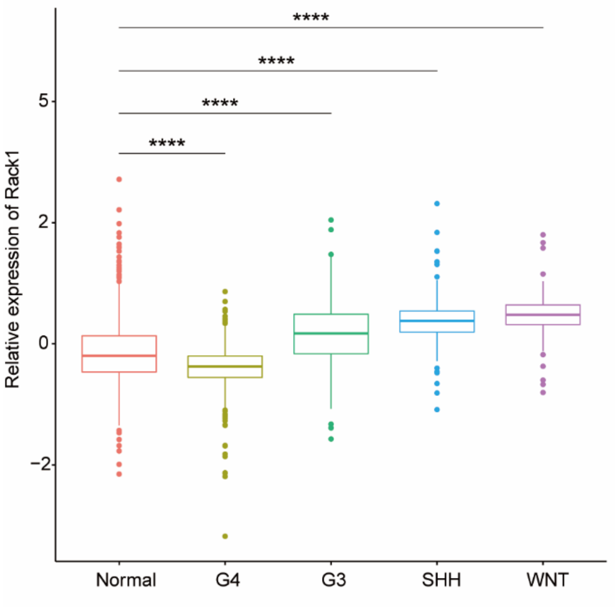

Fig. S4 Relative expression of Rack1 in four types of MB samples compared to normal cerebellum tissue. The expression of Rack1 in different subtypes of MB tumors and normal tissue were analyzed by gene profiling using Gene Expression Omnibus (GEO) DataSet (GSE124814) derived from RNA sequencing data. Data was shown as mean ± SEM; ****$p<0.0001$; Normal, n = 291; G4, n = 530; G3, n = 233; SHH, n = 405; WNT, n = 118. MB: Medulloblastoma; G3: Group 3 subtype; G4: Group 4 subtype; SHH: Sonic Hedgehog.
