# Supplementary material for: Disruption of rack1 suppresses SHH‐type medulloblastoma formation in mice
Source: CNS Neurosci Ther. 2021 Sep 4;27(12):1518–30. doi: 10.1111/cns.13728 (PMC8611787; doi:10.1111/cns.13728)

PC: Positive Control

WT: Rack1$^{F/F}$

MR: Atoh1-Cre; Rack1$^{F/F}$

MS: Atoh1-Cre; SmoM2$^{+/-}$

MSR: Atoh1-Cre; SmoM2$^{+/-}$; Rack1$^{F/F}$

LC3:

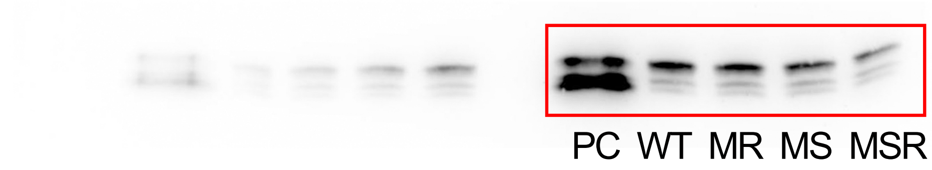

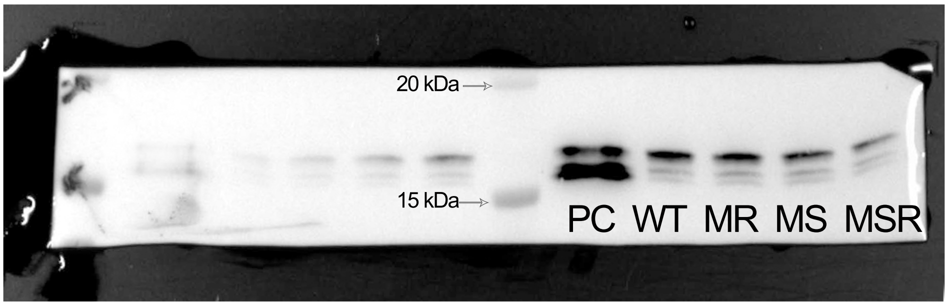

β-actin:

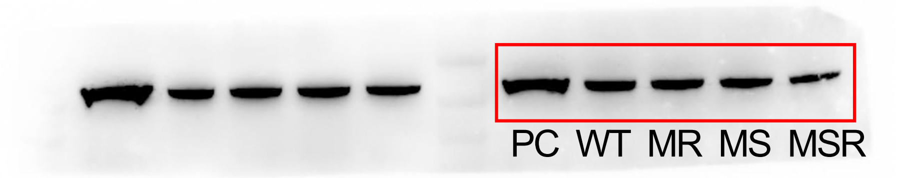

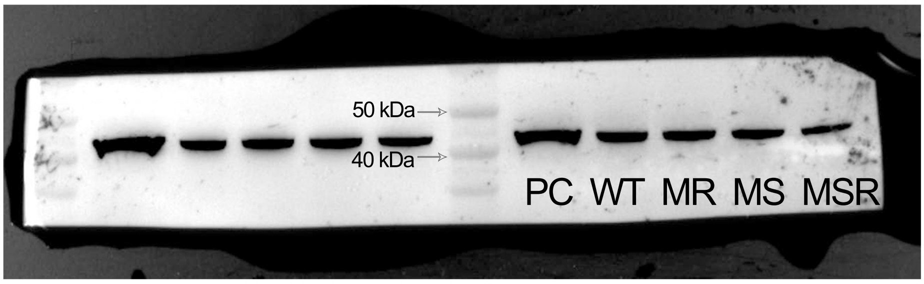

Supplement: Supplementary file 2 — Fig S4 [file CNS-27-1518-s003.pdf]
