# Supplementary material for: Disruption of rack1 suppresses SHH‐type medulloblastoma formation in mice
Source: CNS Neurosci Ther. 2021 Sep 4;27(12):1518–30. doi: 10.1111/cns.13728 (PMC8611787; doi:10.1111/cns.13728)

PC: Positive Control

WT: $SmoM2^{+/-}$;$Rack1^{F/F}$

MR: Atoh1-Cre; $Rack1^{F/F}$

MS: Atoh1-Cre; $SmoM2^{+/-}$

MSR: Atoh1-Cre; $SmoM2^{+/-}$; $Rack1^{F/F}$

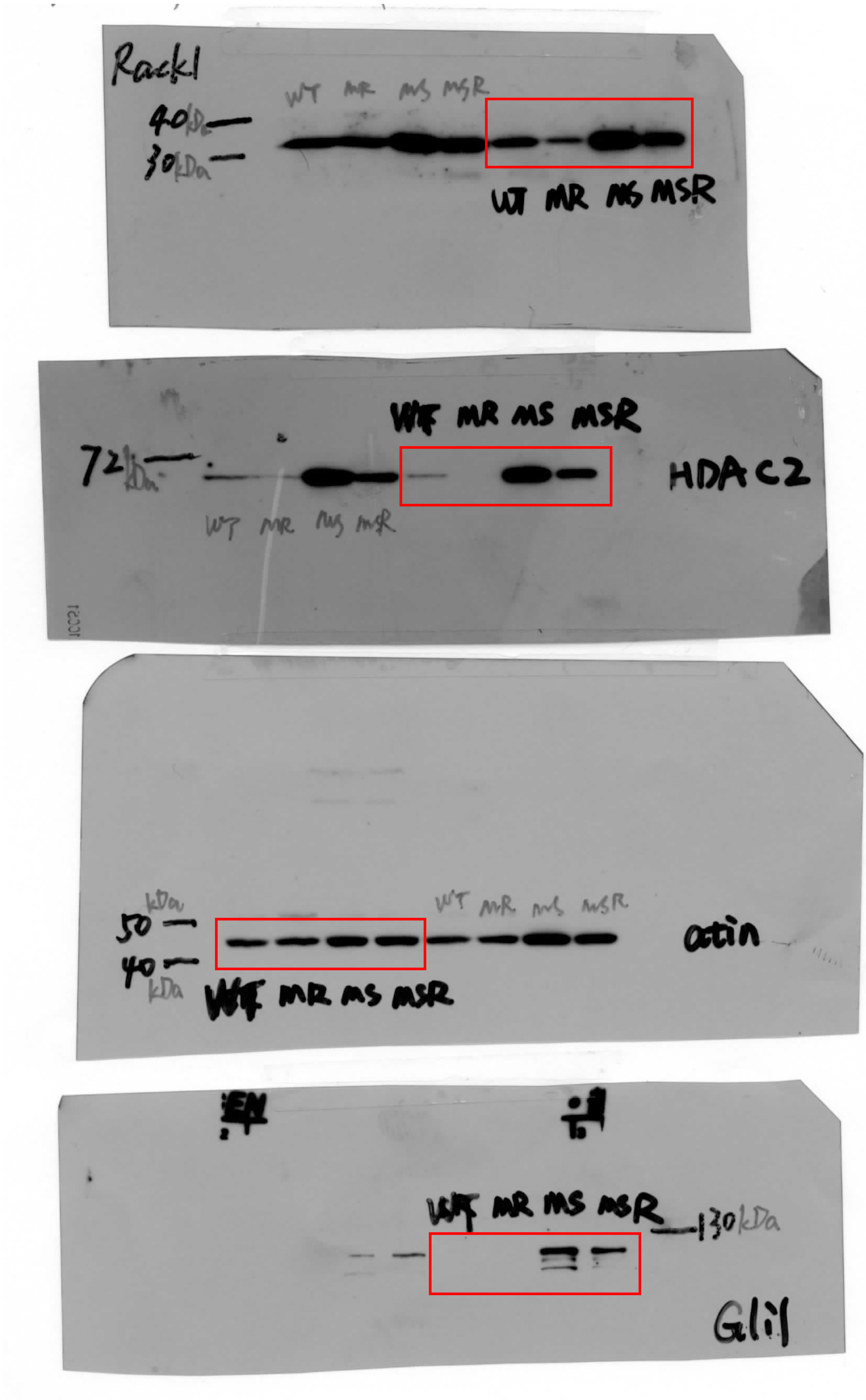

Supplement: Supplementary file 3 — Fig S5 [file CNS-27-1518-s002.pdf]
